# Metabolic and Molecular Changes of the Phenylpropanoid Pathway in Tomato (*Solanum lycopersicum*) Lines Carrying Different *Solanum pennellii* Wild Chromosomal Regions

**DOI:** 10.3389/fpls.2016.01484

**Published:** 2016-10-04

**Authors:** Maria Manuela Rigano, Assunta Raiola, Teresa Docimo, Valentino Ruggieri, Roberta Calafiore, Paola Vitaglione, Rosalia Ferracane, Luigi Frusciante, Amalia Barone

**Affiliations:** ^1^Department of Agricultural Sciences, University of Naples Federico IINaples, Italy; ^2^Istituto di Bioscienze e BioRisorse, UOS Portici, Consiglio Nazionale delle RicercheNaples, Italy

**Keywords:** phenolic acids, chlorogenic acid, flavonoids, pyramided lines, introgression lines

## Abstract

*Solanum lycopersicum* represents an important dietary source of bioactive compounds including the antioxidants flavonoids and phenolic acids. We previously identified two genotypes (IL7-3 and IL12-4) carrying loci from the wild species *Solanum pennellii*, which increased antioxidants in the fruit. Successively, these lines were crossed and two genotypes carrying both introgressions at the homozygous condition (DHO88 and DHO88-SL) were selected. The amount of total antioxidant compounds was increased in DHOs compared to both ILs and the control genotype M82. In order to understand the genetic mechanisms underlying the positive interaction between the two wild regions pyramided in DHO genotypes, detailed analyses of the metabolites accumulated in the fruit were carried out by colorimetric methods and LC/MS/MS. These analyses evidenced a lower content of flavonoids in DHOs and in ILs, compared to M82. By contrast, in the DHOs the relative content of phenolic acids increased, particularly the fraction of hexoses, thus evidencing a redirection of the phenylpropanoid flux toward the biosynthesis of phenolic acid glycosides in these genotypes. In addition, the line DHO88 exhibited a lower content of free phenolic acids compared to M82. Interestingly, the two DHOs analyzed differ in the size of the wild region on chromosome 12. Genes mapping in the introgression regions were further investigated. Several genes of the phenylpropanoid biosynthetic pathway were identified, such as one *4-coumarate:CoA ligase* and two *UDP-glycosyltransferases* in the region 12-4 and one *chalcone isomerase* and one *UDP-glycosyltransferase* in the region 7-3. Transcriptomic analyses demonstrated a different expression of the detected genes in the ILs and in the DHOs compared to M82. These analyses, combined with biochemical analyses, suggested a central role of the 4-coumarate:CoA ligase in redirecting the phenylpropanoid pathways toward the biosynthesis of phenolic acids in the pyramided lines. Moreover, analyses here carried out suggest the presence in the introgression regions of novel regulatory proteins, such as one Myb4 detected on chromosome 7 and one bHLH detected in chromosome 12. Overall our data indicate that structural and regulatory genes identified in this study might have a key role for the manipulation of the phenylpropanoid metabolic pathway in tomato fruit.

## Introduction

**FIGURE 1:**
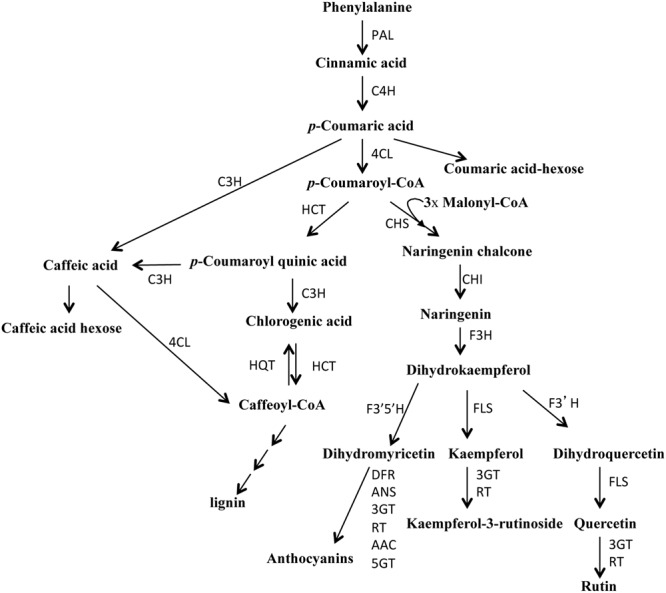
**Schematic overview of the phenylpropanoid pathway in tomato.** PAL, phenylalanine-ammonia-lyase; C4H, cinnamate 4-hydroxylase; 4CL, 4-coumarate:CoA ligase; HCT, cinnamoyl-CoA shikimate/quinate transferase; C3H, *p*-coumaroyl ester 3-hydroxylase; HQT, hydroxycinnamoyl-CoA quinate transferase; CHS, chalcone synthase; CHI, chalcone isomerase; F3H, flavanone-3-hydroxylase; F3′H, flavonoid-3′-hydroxylase; FLS, flavonol synthase; 3GT, flavonoid-3-*O*-glucosyltransferase; RT, flavonoid 3-*O*-glucoside-rhamnosyltransferase; F3′5′H, flavonoid-3′-5′-hydroxylase; DFR, dihydroflavonol reductase; ANS, anthocyanidin synthase; AAC, anthocyanin acyltransferase; 5GT, flavonoid 5-glucosyltransferase.

Tomato (*Solanum lycopersicum*) is the second most consumed vegetable in the world; indeed, tomato consumption reaches 40–45 kg *pro capita per* year in several European countries (FAO database). Consumption of tomato fruits is associated with a reduced risk of some types of cancer and of several chronic non-communicable diseases (CNCDs), such as diabetes, hypertension, and obesity (Raiola et al., 2014). These health benefits are mainly attributed to the occurring of hydrophilic and lipophilic phytochemicals (polyphenols, ascorbic acid, carotenoids, and tocopherols) in the fruits. Among these, polyphenols are very active compounds that in humans are able to reduce DNA oxidation and to control inflammation and cell proliferation and differentiation (Lodovici et al., 2001; Visioli et al., 2011). In plants these secondary metabolites are implicated in UV-B tolerance, plant response toward biotic and abiotic stimuli, growth control and developmental processes (Vogt, 2010; Tohge et al., 2015). In the first step of the general phenylpropanoid biosynthetic pathway, the phenylalanine is deaminated by the enzyme PAL (phenylalanine ammonia lyase) to form cinnamic acid that is then hydroxylated to generate coumaric acid (**Figure 1**). The enzyme 4-coumarate:CoA ligase (4CL) catalyzes the last step of the general phenylpropanoid pathway. The enzyme 4CL converts coumaric acid and other substituted cinnamic acids (caffeic, ferulic, and sinapic acids) into corresponding CoA esters that are then used for the biosynthesis of flavonoids, isoflavonoids, lignins, coumarins, and other phenolics (Alberstein et al., 2012; Sun et al., 2013; Li et al., 2015; Pandey et al., 2015). It is thought that the substrate specificity of 4CL determines the direction of the metabolic flux in the downstream reactions (Alberstein et al., 2012). In tomato, flavonoids are located mostly in the skin and are involved in the pigmentation and aroma of the fruit; they include naringenin, quercetin, rutin, kampferol, and catechin and show a protective action against intestinal inflammation and rheumatoid arthritis (Kauss et al., 2008; González et al., 2011; Raiola et al., 2014). Phenolic acids are responsible for the astringent taste of tomato fruits and consist mainly of gallic, chlorogenic, and ferulic acids (Moco et al., 2007). Hydroxycinnamates, due to their antioxidant capacity, have important beneficial health effects: they can limit LDL (low-density lipid) oxidation, prevent carcinogenesis and are potential therapeutic agents for neurodegenerative diseases, such as Alzheimer and Parkinson and for the prevention of cardiovascular disease and diabetes (Niggeweg et al., 2004; Calvenzani et al., 2015; Tohge et al., 2015).

The cultivated tomato varieties generally do not contain high amounts of phenolic compounds in the fruit (Tohge et al., 2015). This is also due to tomato domestication that resulted in the loss of about 95% of the chemical diversity of wild relatives (Perez-Fons et al., 2014). For example, domestication in *S. lycopersicum* has led to poor tasting tomatoes also due to reduced formation of volatile compounds (Bolger et al., 2014). Several strategies have been previously used to increase the content of antioxidants in tomato fruits. One strategy considers screening wild genetic resources for quality traits, such as antioxidant content, that could be introduced into modern varieties (Gur and Zamir, 2004; Schauer et al., 2006). Around 20 years ago nearly isogenic lines were generated to effectively reintroduce unused genetic variation from wild species into cultivated varieties and to facilitate the mapping of traits originating from wild donors (Gur and Zamir, 2015). Introgression lines (ILs) include single marker-defined introgressed genomic regions from the wild species into the genomic background of the cultivated variety *S. lycopersicum* (M82). *Solanum pennellii* ILs were produced and were used to map several QTLs associated with traits related to tomato fruit quality (Eshed and Zamir, 1995; Rousseaux et al., 2005). We previously identified two introgression lines (IL7-3 and IL12-4) carrying loci from the wild species *S. pennellii* that increase antioxidants in the fruit (Sacco et al., 2013). Successively, these lines were crossed and genotypes carrying both introgressions at the homozygous condition were selected (Rigano et al., 2014). When we examined their nutritional quality we found that the amount of total antioxidant compounds was increased in the pyramided lines compared to the parental lines and the cultivated control genotype M82. Additional metabolic analyses revealed significant increase of total polyphenols in the pyramided lines compared to the parental lines and to M82 and a concomitant reduction of flavonoids (Rigano et al., 2014). In this study, two pyramided lines with a different *S. pennellii* introgression region in chromosome 12 were selected and analyzed in order to better investigate the genetic mechanisms underlying the interaction between the two wild regions. The integration of genomic, transcriptomic, metabolic and biochemical analyses was carried out and allowed us to define the role of different wild *S. pennellii* genes in redirecting the phenylpropanoid pathways toward the biosynthesis of phenolic acids in the pyramided lines.

## Materials and Methods

### Chemical and Reagents

Phenylalanine, cinnamic, ferulic, caffeic, *p*-coumaric, chlorogenic and gallic acids, rutin, and quercetin standard were purchased from Sigma (Italy), naringenin from Aldrich (Italy), naringenin-7-*O*-glucoside from Infodine (USA). Methanol, formic acid, and water HPLC grade were obtained from Merck (Darmstadt, Germany). Deionized water was obtained from a Milli-Q water purification system (Millipore, Bedford, MA, USA). Chromatographic solvents were degassed for 20 min using a Branson 5200 (Branson Ultrasonic, Corp., USA) ultrasonic bath.

### Plant Material and Growth Conditions

Seeds from IL12-4 (LA4102), IL7-3 (LA4066) and their parental line M82 (LA3475) were kindly provided by the Tomato Genetics Resource Centre (TGRC)^1^. Genotypes DHO88 and DHO88-SL were selected from F_2_ genotypes previously obtained by intercrossing IL12-4 and IL7-3 (Sacco et al., 2013). The F_2_ genotypes were selfed for two generations and then screened by species-specific markers. During the years 2014 and 2015, the double-homozygous plants of the F_4_ progenies and their parents were grown in an experimental field located in Acerra (Naples, Italy), according to a completely randomized design with three replicates (10 plants/replicate). The physico-chemical properties of the soil have been reported in Supplementary Table S1. Seeds were first germinated in Petri dishes on water-soaked filter paper and subsequently transferred in peat on a seed tray and incubated in a growth chamber at 22°C and 16 h/8 h light/dark. Plants were transplanted at the four leaf-stage. Before transplanting urea phosphate fertilizer (40 kg ha^-1^) was applied to the soil. Tillage treatments included plowing followed by one or two milling. Successively, weeding and ridging were carried out. Plants were irrigated as required (2–3 times *per* week in absence of rain). Recommended levels of N (190 kg ha^-1^), P (25 kg ha^-1^), and K (20 kg ha^-1^) were applied during cultivation *via* fertirrigation. During the growing season, the insecticides and fungicides were applied according to general local practices and recommendations. In the two growing seasons we recorded temperatures and precipitation in the seasonal media for the Campania region, even though in 2014 rainfall was slightly heavier than in 2015, whereas in the latter year the temperatures where slightly higher than in 2014.

Samples of about 20 full mature red fruits *per* plot were collected. Tomato fruits were chopped, ground in liquid nitrogen in a blender (FRI150, Fimar) to a fine powder, and kept at -80°C until the subsequent metabolic, molecular, and enzymatic analyses were performed.

### Chemical Extractions

For the metabolic analyses, each sample consisted of 20-pooled fruits *per* plot. The extraction of the polyphenolic fraction was carried out according to the procedure reported by Choi et al. (2011) with some changes. Briefly, frozen tomato powder (3 g) was weighed, placed into a 50 ml Falcon tube, and extracted with 15 ml of 70% methanol into an ultrasonic bath (Branson 5200, Ultrasonic, Corp.) for 30 min at 30°C. The mixture was centrifuged at 20000 *g* for 10 min at 4°C, and the supernatant was collected, while the pellet was re-extracted for the second time as previously described. An aliquot (500 μl) of the methanolic extract was stored at -20°C until further analyses, while 25 ml of extract were dried by rotary evaporator (Buchi R-210, Milan, Italy) at 30°C for 10 min and dissolved in 70% methanol (2 ml). Then, the extract was transferred in a glass tube and was further dried by using a SpeedVac (Thermo Scientific, Savant, SPD131DDA SpeedVac Concentrator, Waltham, MA, USA). The dried extract was dissolved in 70% methanol (500 μl) obtaining a final concentration of 5 g fresh weight (FW)/ml. The extract was passed through a 0.45 μm Millipore nylon filter (Merck Millipore, Bedford, MA, USA) and stored at -20°C until LC/MS/MS analysis.

### Total Flavonoids

Total flavonoids were quantified by the aluminum chloride colorimetric test reported by Marinova et al. (2005) with slight modifications. An aliquot (500 μl) of methanolic extract (see Chemical Extractions) was added to 5% NaNO_2_ (30 μl) and, after an incubation of 5 min, 10% AlCl_3_ (30 μl) was added. After 6 min 1 M NaOH (200 μl) and H_2_O (240 μl) were added and the absorbance of the resulting solution was measured at 510 nm. Total flavonoids content was expressed as mg quercetin equivalent (QE)/100 g FW. Three biological replicates and three technical assays for each biological repetition were analyzed.

### LC/MS/MS Analysis of Polyphenols

Chromatographic separation was performed using an HPLC apparatus equipped with two Micropumps Series 200 (PerkinElmer, Shellton, CT, USA), a UV/VIS series 200 detector (PerkinElmer, Shellton, CT, USA) set at 330 nm and a Prodigy ODS3 100 Å column (250 mm × 4.6 mm, particle size 5 μ; Phenomenex, CA, USA).

The eluents were: A water 0.2% formic acid; B aceto nitrile/methanol (60:40, v/v). The gradient program was as follows: 20–30% B (6 min), 30–40% B (10 min), 40–50% B (8 min), 50–90% B (8 min), 90–90% B (3 min), 90–20% B (3 min) at a constant flow of 0.8 ml/min. The LC flow was split and 0.2 ml/min was sent to the mass spectrometry. Injection volume was 20 μl. Mass spectrometer analyses were performed on an API 3000 triple quadrupole (Applied Biosystems, Canada) equipped with a TurboIonSpray source working in the negative ion mode. The analyses were performed in MRM (multiple reaction monitoring), using the following settings: drying gas (air) was heated to 400°C, capillary voltage (IS) was set to 4000 V. The MS/MS characteristics of phenolic compounds identified in extracts are reported in Supplementary Table S2. Example of a chromatogram of phenolic compounds in M82 detected at 330 nm is reported in Supplementary Figure S1.

The compounds were identified comparing retention times and MS/MS fragments with standards data. Identification of compounds that were not available as standards was obtained comparing their MS and MS/MS spectra with the literature data (Moco et al., 2006; Vallverdú-Queralt et al., 2011).

### Molecular Marker Analyses

In order to define the wild region size of the DHO lines, polymorphic markers previously selected in our laboratory and spanning the introgression regions 7-3 and 12-4 were used (Ruggieri et al., 2015; Calafiore et al., 2016). Total genomic DNA was extracted from leaves using the PureLink^TM^ Genomic DNA Kit (Invitrogen). PCR DNA amplification was carried out in 50 μl reaction volume containing 50 ng DNA, 1X reaction buffer, 0.2 mM each dNTP, 1.0 mM primer and 1.25 U GoTaq polymerase (Promega). The restriction endonuclease reaction was performed in 50 μl of reaction volume containing 20 μl PCR product, 5 μl 10X reaction buffer and 1 μl of the selected restriction enzyme (10 u/ml). Digested fragments were separated by electrophoresis on 2% agarose gel in 1X TAE buffer.

### Identification and Expression of Candidate Genes

The search for candidate genes (CG) mapping in the regions 7-3 and 12-4 of chromosomes 7 and 12 and potentially associated with phenolics metabolism was conducted by exploring the annotations and the Gene Ontology terms of genes included in the two regions. The number of CGs was then reduced by selecting only those expressed in the fruit at different developmental stages in the reference cv. Heinz, as reported in the Tomato Functional Genomic Database (TED^2^). RNA-Seq data from the red fruit of *S. pennellii* ILs and of *S. lycopersicum* cv. M82 were also retrieved from the TED.

The expression of CGs in the ILs fruit compared to that in M82 was verified by Real-Time PCR amplification. Total RNA was isolated from tomato fruit of lines M82, IL7-3, IL12-4, DHO88, and DHO88-SL by using the TRIzol^®^ reagent (Invitrogen, Carlsbad, CA, USA) and treated with RNase-free DNase (Invitrogen, Carlsbad, CA, USA; Madison, WI, USA) according to the method reported by the manufacturer (Invitrogen). Total RNA (1 μg) was treated by the Transcriptor High Fidelity cDNA Synthesis Kit (Roche) and cDNA was stored at -20°C until RT-PCR analysis. For each RT-PCR reaction, 1 μl of cDNA diluted 1:10 was mixed with 12.5 μl SYBR Green PCR master mix (Applied) and 5 pmol each of forward and reverse primers (Supplementary Table S3) in a final volume of 25 μl. The reaction was carried out by using the 7900HT Fast-Real Time PCR System (Applied Biosystems). The amplification program was carried out according to the following steps: 2 min at 50°C, 10 min at 95°C, 0.15 min at 95°C and 60°C for 1 min for 40 cycles. In order to verify the amplification specificity, the amplification program was followed by the thermal denaturing step (0.15 min at 95°C, 0.15 min at 60°C, 0.15 min at 95°C) to generate the dissociation curves. All reactions were run in triplicate for each of the three biological replicates and a housekeeping gene coding for the *elongation factor 1-alpha* (*Ef 1- α* – Solyc06g005060) was used as reference gene (Calafiore et al., 2016). The expression levels relative to the reference gene were calculated using the formula 2^-ΔCT^, where ΔCT = (CT _RNAtarget_ – CT _reference RNA_) (Schmittgen et al., 2004). Comparison of RNA expression was based on a comparative CT method (ΔCT) and the relative expression was quantified and expressed according to log_2_RQ, where RQ was calculated as 2^-ΔΔCT^ and where ΔCT = (CT _RNAtarget_ – CT _reference RNA_) – (CT _calibrator_ – CT _reference RNA_) (Winer et al., 1999; Livak and Schmittgen, 2001). M82 was selected as calibrator. Quantitative results were expressed as the mean value ± SE.

### Phylogenetic Analysis

All known and reported 4CL and UDP-glycosyltransferase protein-coding sequences were retrieved from the National Center for Biotechnology Information (NCBI). In total, 34 4CL protein sequences and 38 UDP-glycosyltransferases from several dicots and monocots species were collected and accession numbers are reported in Supplementary Table S4. The 4CL and UDP amino acid alignments were performed using ClustalW implemented in MEGA 6 (Tamura et al., 2013) and non-rooted phylogenetic trees were constructed using the Maximum Likelihood method and the Jones-Taylor-Thornton (JTT) model using default parameters. Initial trees for the heuristic search were obtained by applying the Neighbor-Joining method to a matrix of pairwise distances estimated using a JTT model. All positions containing gaps and missing data were eliminated. Bootstrap-supported consensus trees were inferred from 500 replicates. Branches with <50% bootstrap support were collapsed.

### Enzymatic Assays

Enzyme extractions were performed at 4°C following the method described in Weitzel and Petersen (2010) with slight modifications. Tomato frozen powder (0.3 g) was ground with 0.1 M potassium phosphate buffer pH 7.5 containing 1 mM DTT, 0.1 mM EDTA, 5 mM ascorbic acid, 1 mM PMSF, 0.15% w/v PVP. Then the homogenate was centrifuged at 12000 *g* for 20 min at 4°C and the supernatant was used as a source of crude enzymes for assaying PAL and 4CL activities. Protein concentration was evaluated by the method of Bradford (1976).

Phenylalanine ammonia lyase activity was determined spectrophotometrically. The reaction mixture contained 50 mM Tris-HCl buffer pH 8.9, 3.6 mM NaCl, 10 mM phenylalanine and 50 μl protein extract. The reaction was incubated at 37°C for 1 h and stopped by adding 150 μl 6 M HCl. The tubes were centrifuged for 10 min at 12000 *g*. The absorbance was read at 290 nm using as control a reaction without phenylalanine. The rate of appearance of cinnamic acid was taken as a measure of enzyme activity using an increase of 0.01 A_290_ equal to 3.09 nmol of cinnamic acid formed (Saunders and McClure, 1975).

4CL enzyme activity was measured spectrophotometrically. The reaction mixture contained 0.1 M potassium phosphate buffer, pH 7.5, 2.5 mM ATP, 2.5 mM MgCl_2_, 1 mM DTT, 50 μl protein preparation and 0.5 mM substrate. The reaction was started by the addition of 0.3 mM CoA and incubated for 1 h at 40°C. The formation of the respective CoA thioesters was measured at different path length depending on the used substrate: 311 nm (cinnamic acid), 333 nm (4-coumaric acid), 346 nm (caffeic acid), and 345 nm (ferulic acid). The extinction coefficient of these esters was used to calculate enzyme activity (Lee et al., 1997; Chen et al., 2006).

### Statistical Analyses

In Real-time q-PCR analyses, differences of expression of CGs among samples were determined by using SPSS (Statistical Package for Social Sciences) Package 6, version 15.0. Significant different expression levels were determined by comparing the genotypes through a Student’s *t*-test at a significance level of 0.05. In metabolic analyses, quantitative results were expressed as the mean value ± SD. Differences among analyzed genotypes were determined by using SPSS (Statistical Package for Social Sciences) Package 6, version 15.0 (SSPS, Inc., Chicago, IL, USA). Significant different metabolite levels were determined by comparing mean values through a factorial analysis of variance (ANOVA) with Duncan *post hoc* test at a significance level of 0.05. Enzymatic data were subjected to ANOVA statistical analyses and means were compared using the Tukey HSD test (*p* ≤ 0.05) by using SigmaPlot software.

The percentage of variations of quantitative parameters compared to M82 was calculated by using the following formula:

$$\begin{array}{c} \mathrm{Increase}\;\mathrm{or}\;\mathrm{Decrease}(\%)\;=\;\left[\frac{value\;of\;tested\;genotype-value\;of\;M82}{value\;of\;M82}\right]*100. \end{array}$$

## Results

### Phenolic Compounds in Introgression and Pyramided Lines

**FIGURE 2:**
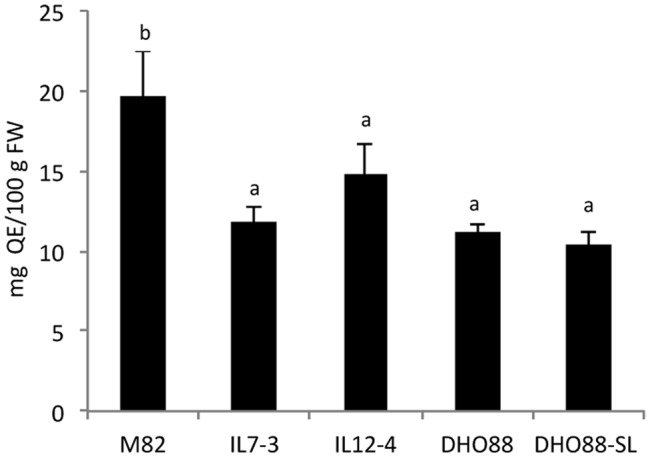
**Content of total flavonoids calculated in red ripe fruits of M82, IL7-3, IL12-4, DHO88, and DHO88-SL.** Total flavonoids are expressed as mg QE/100 g FW. Values are mean ± SD. Values with different letters are significantly different (*p* < 0.05).

Metabolic analyses were performed on mature red fruits of the cultivated genotype M82, of the ILs 7-3 and 12-4 and of two selected pyramided lines (DHO88 and DHO88-SL) obtained by crossing the two introgression lines (IL7-3 × IL12-4). The cultivated genotype M82 contained a mean concentration of flavonoids of 19.70 ± 2.74 mg/100 g FW that was reduced by 40.2% in IL7-3 and by 25.1% in IL12-4 (**Figure 2**). A significant decrease of total flavonoids in the pyramided genotypes compared to the cultivated genotype M82 was also recorded and was comparable to that calculated in the parental lines IL7-3 and IL12-4.

**FIGURE 3:**
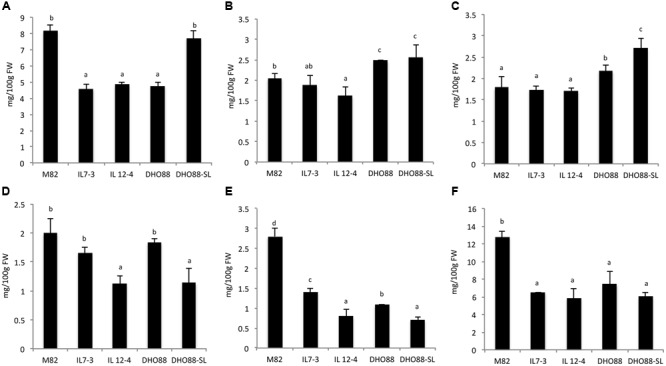
**Phenolic compounds amount (mg/100 g FW) calculated in red ripe fruits from M82, IL7-3, IL12-4, DHO88, and DHO88-SL quantified by LC/MS/MS: **(A)** chlorogenic acid; **(B)** coumaric acid hexose; **(C)** caffeic acid hexose; **(D)** rutin; **(E)** naringenin glucoside; **(F)** chalconaringenin.** Values are mean ± SD. Values with different letters are significantly different (*p* < 0.05).

Results from LC/MS/MS analysis of polyphenols are reported in Supplementary Table S5 and **Figure 3**. Data showed that chlorogenic acid, coumaric acid hexose, caffeic acid hexose, rutin, naringenin glucoside, and chalconaringenin were the main polyphenols present in all the samples. Considering the parental lines, the amount of chlorogenic acid, the most abundant compound among free phenolic acids in the analyzed lines, significantly decreased both in IL7-3 (-43.6%), and in IL12-4 (-40.5%) compared to M82. A decrease of caffeic acid in IL12-4 (-31.25%) compared to M82 was also detected (Supplementary Table S5). Regarding the fraction of hexoses, the amount of coumaric acid hexose decreased in IL12-4 compared to M82, while the concentrations of caffeic acid hexose were comparable in both the ILs and in M82. The amount of detected flavonoids was significantly different in the genotypes analyzed. In particular, the compound rutin decreased of 43.5% in IL12-4 compared to M82. A significant decrease of naringenin glucoside and of chalconaringenin was detected both in IL7-3 and in IL12-4 compared to M82.

As for the pyramided lines, the amount of chlorogenic acid exhibited a significant decrease (-41.5%) only in DHO88 compared to M82. By contrast, this acid significantly increased in DHO88-SL compared to the ILs and to the pyramided line DHO88. As for the content of caffeic acid, no significant differences were detected in DHO88 compared to M82, whereas a significant increase was found in DHO88-SL compared to the cultivated line and to the parental lines. Both coumaric acid hexose and caffeic acid hexose were significantly higher in DHO88 and DHO88-SL than in M82 and in the ILs. Overall, in the DHO lines the content of phenolic acids increased compared to the parental line IL7-3 and IL12-4, above all the fraction of hexoses.

As for the flavonoids, the amount of rutin detected in DHO88 was comparable to that found in M82 and IL7-3. In contrast, in DHO88-SL it decreased compared to M82 and was comparable to the amount recorded in IL12-4. Lower levels of naringenin glucoside and chalconaringenin were found in both the pyramided lines compared to M82. The amount of chalconaringenin detected in the pyramided lines was comparable to the amount found in the ILs.

### Genomic Characterization of DHO Lines

In order to understand which genetic mechanisms might explain the interactions between the two wild *S. pennellii* regions pyramided in DHO genotypes in influencing the phenylpropanoid metabolism, the introgression regions borders were precisely defined by using molecular markers reported in Ruggieri et al. (2015) and Calafiore et al. (2016) (**Table 1**). In both genotypes DHO88 and DHO88-SL the wild region 7-3 stretches from marker N27 to marker N17, spanning the same 6.6 Mbp region of IL7-3. By contrast, the wild region of chromosome 12 has different size in DHO88 and DHO88-SL. In particular, in DHO88 this stretches from marker M1 to marker M18, whereas from marker M10 to marker M18 in the line DHO88-SL, thus reducing in the latter the *S. pennellii* genome to 2.1 Mbp. Consequently, the number of wild alleles at potential CGs for phenylpropanoid accumulation varied in the two lines. Out of 725 genes mapping in the region 7-3 (Calafiore et al., 2016), four CGs involved in the flavonoid biosynthetic pathway were identified, that are Solyc07g062030 annotated as a chalcone-flavonone isomerase (CHI) and three genes coding for UDP-glucosyltranferase (UGT). Out of 480 genes mapping in the region 12-4 (Ruggieri et al., 2015), DHO88 line shares with IL12-4 the same wild alleles for 14 CGs involved in the phenylpropanoid metabolism, that are one *4-coumarate:CoA ligase* (*4CL*), seven *UDP-glucosyltransferase*, three *hydroxycinnamoyl-CoA quinate transferase* (*HCT*), one *N-hydroxycinnamoyl/benzoyltransferase*, one *N-acetyltransferase*, and one *chalcone synthase*. Due to its reduced introgression region size, line DHO88-SL included wild alleles for 13 CGs, the most consistent difference between DHO88 and DHO88-SL being the lack of the *4-coumarate:CoA ligase* wild allele in line DHO88SL. Interestingly, all the genes for the biosynthesis of flavonoids were located in the lower part of the introgressed region 12-4. In addition, several transcription factors (TFs), such as the TFs Myb, WD-40 and bHLH, were identified in both introgressed regions. Among the identified TFs, three Myb like-4 mapped in the introgressed region 7-3. Myb4 TFs are known to be able to negatively regulate the expression of several genes of the phenylpropanoids pathway such as *cinnamate 4-hydrolase* and *dihydroflavonol 4-reductase* (Preston et al., 2004). Out of the 37 CGs and TFs identified in the introgressed regions 7-3 and 12-4, 28 genes are not expressed in tomato fruit, as reported in the Tomato Functional Genomics Database (**Table 1**). These genes were eliminated from subsequent analyses.

**Table 1:** Candidate genes mapping in the introgressed regions 7-3 and 12-4.

Candidate gene	Gene identifier (Solyc ID)	Gene position (release SL2.50) bp	Expression level (RPKM)
**Chromosome 7**			
**N27**			
Myb family transcription factor-like	Solyc07g049640	59978797–59979255	0.00
Myb-related transcription factor	Solyc07g052300	60797135–60800394	0.00
Myb family transcription factor	Solyc07g052490	61003154–61004234	0.00
Glycosyltransferase-like protein	Solyc07g052630	61096621–61100859	0.00
Glycosyltransferase-like protein	Solyc07g052650	61109726–61111909	0.00
Transcription factor (Fragment)	Solyc07g052670	61115970–61120548	0.55
bHLH transcription factor-like	Solyc07g052930	61324832–61326734	0.30
Myb like-4	Solyc07g053230	61699380–61700488	1.97
Myb like-4	Solyc07g053240	61710088–61711189	0.18
Myb like	Solyc07g053630	62062717–62066811	0.00
Myb transcription factor	Solyc07g054840	63019149–63020392	0.00
Myb-related transcription factor	Solyc07g054960	63121625–63123021	0.00
Myb-related transcription factor	Solyc07g054980	63133145–63137020	0.00
Myb like-4	Solyc07g055000	63145852–63147956	0.36
Glycosyltransferase	Solyc07g055930	63851843–63858435	38.33
Myb transcription factor	Solyc07g056120	63983586–63986633	5.06
Chalcone–flavonone isomerase	Solyc07g062030	64874570–64877582	6.41
Transcription factor bHLH126	Solyc07g062200	64999668–65002971	0.00
**N17**			
**Chromosome 12**			
**M1**			
4-Coumarate-CoA ligase	Solyc12g094520	64739117–64737891	26.05
**M10**			
UDP-glucosyltransferase family 1 protein	Solyc12g096080	65151226–65150412	0.00
N-hydroxycinnamoyl/benzoyltransferase 5	Solyc12g096250	65264285–65263439	0.06
Hydroxycinnamoyl CoA quinate transferase	Solyc12g096770	65547985–65549310	0.00
Hydroxycinnamoyl CoA quinate transferase	Solyc12g096790	65568543–65567197	0.00
Hydroxycinnamoyl CoA quinate transferase	Solyc12g096800	65570588–65569209	0.00
UDP-glucosyltransferase family 1 protein	Solyc12g096820	65586813–65587528	0.25
UDP-glucosyltransferase family 1 protein	Solyc12g096830	65589413–65590134	8.56
N-acetyltransferase	Solyc12g096840	65599970–65599549	0.00
UDP-glucosyltransferase 1	Solyc12g096870	65622881–65624341	0.10
Chalcone synthase	Solyc12g098090	65743943–65744925	0.00
UDP-glucosyltransferase family 1 protein	Solyc12g098580	66045023–66043754	1.02
UDP-glucosyltransferase family 1 protein	Solyc12g098590	66047542–66048121	0.00
UDP-glucosyltransferase family 1 protein	Solyc12g098600	66049474–66050865	0.00
bHLH	Solyc12g098620	66065713–66066266	5.95
WD-repeat protein-like	Solyc12g098690	66118366–66118781	36.47
Myb transcription factor	Solyc12g099120	66392195–66392661	0.15
Myb transcription factor	Solyc12g099130	66395992–66396421	0.38
Myb transcription factor	Solyc12g099140	66404232–66404530	0.56
**M18**			

*For each Solyc the position on the chromosome is reported in bp. RPKM: expression values as those reported in the Tomato Functional Genomic Database.*

### Expression Variability of Selected Candidate Genes

**FIGURE 4:**
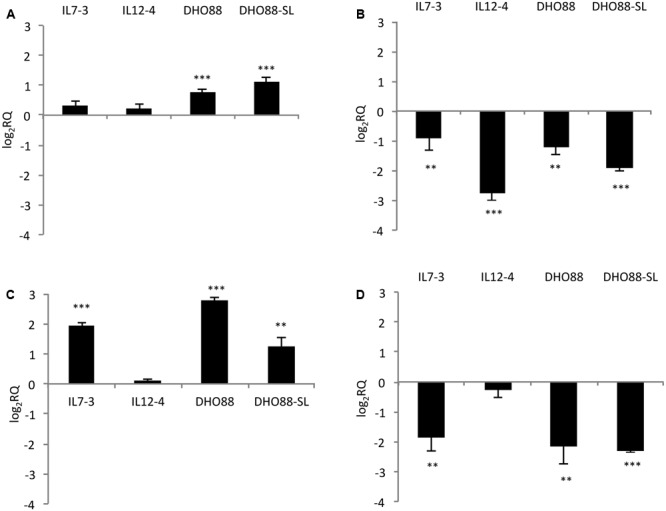
**Real-time qPCR analysis of expression of the CGs mapping in the introgressed region 7-3 in the fruit of IL7-3, IL12-4, DHO88, and DHO88-SL.** The expression levels of **(A)** Solyc07g062030 (CHI); **(B)** Solyc07g055930 (UGT); **(C)** Solyc07g053230 (Myb 4-like); **(D)** Solyc07g056120 (Myb) are reported in comparison to those observed in M82. Asterisks indicate statistically significant differences of each genotype compared to M82 (^∗∗^*p* < 0.01, ^∗∗∗^*p* < 0.001).

We studied the modulation in expression of nine selected CGs in ripe fruits of M82, ILs and pyramided lines DHO88 and DHO88-SL through real-time q-PCR. As for the introgressed region 7-3, we analyzed the expression of two selected genes involved in the biosynthetic pathways and of two genes coding for TFs (**Figure 4**). The gene coding for one chalcone isomerase (CHI – Solyc07g062030) demonstrated a higher expression only in the lines DHO88 and DHO88-SL compared to M82. A drop in the expression of the gene coding for one UDP-glucosyltransferase (UGT- Solyc07g055930) was demonstrated in all the genotypes tested compared to M82. The expression of the gene coding for the TF Myb4-like Solyc07g053230 was higher in IL7-3 and in the two pyramided lines compared to M82. Finally, the gene coding for the Myb Solyc07g056120 showed a lower expression level in IL7-3 and in the two pyramided lines compared to M82.

**FIGURE 5:**
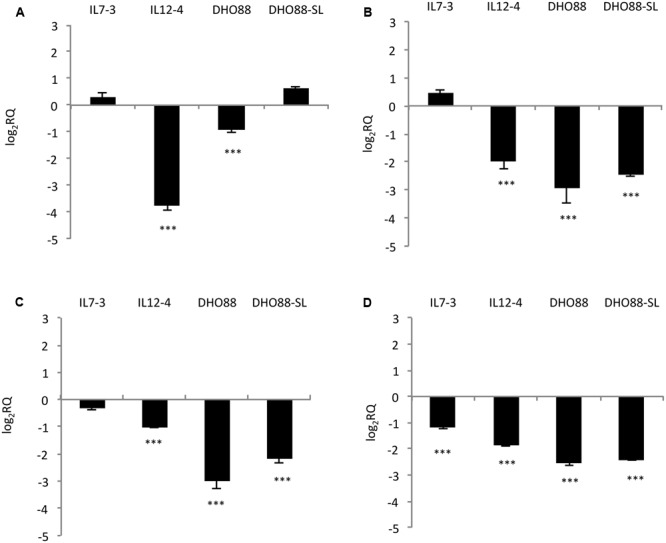
**Real-time qPCR analysis of expression of the CGs mapping in the introgressed region 12-4 in the fruit of IL7-3, IL12-4, DHO88, and DHO88-SL.** The expression levels of **(A)** Solyc12g094520 (4CL); **(B)** Solyc12g098580 (UGT); **(C)** Solyc12g098620 (bHLH); **(D)** Solyc12g098690 (WD40) are reported in comparison to those observed in M82. Asterisks indicate statistically significant differences of each genotype compared to M82 (^∗∗∗^*p* < 0.001).

As for genes mapping on the introgressed region 12-4 (**Figure 5**), the gene Solyc12g094520 coding for one 4-coumarate:CoA ligase and located in the upper part of the introgression region 12-4 displayed lower mRNA levels in ripe fruits of IL12-4 and DHO88 compared to M82. The expression of the gene Solyc12g096830 coding for one UDP-glucosyltransferase (UGT) did not change in the lines here tested (data not shown). A drop in expression of the gene Solyc12g098580 coding for another UDP-glucosyltransferase family 1 (UGT) protein and located in the lower part of the introgressed region 12-4 was instead recorded in IL12-4 and in both the pyramided lines. Interestingly, a down-regulation of the gene Solyc12g098620 coding for one bHLH protein and of the gene Solyc12g098690 coding for one WD40 protein was recorded in IL12-4 and in both pyramided lines. A lower expression of the gene Solyc12g098690 was recorded also in the introgression line IL7-3. Interestingly, the genes coding for the TFs bHLH and WD40 were located next to the gene Solyc12g098580 coding for one UGT.

**FIGURE 6:**
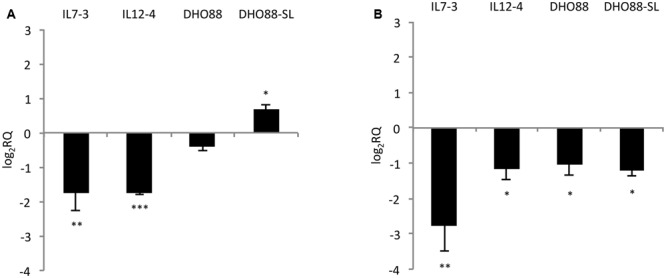
**Real-time qPCR analysis of expression of the genes *HQT* and *Myb12* in the fruit of IL7-3, IL12-4, DHO88 and DHO88-SL.** The expression levels of **(A)** Solyc07g005760 (HQT) and **(B)** Solyc01g079620 (Myb12) are reported in comparison to those observed in M82. Asterisks indicate statistically significant differences of each genotype compared to M82 (^∗^*p* < 0.05, ^∗∗^*p* < 0.01, ^∗∗∗^*p* < 0.001).

Additionally, we tested the expression of two genes located outside of the introgressed regions (**Figure 6**). We tested the expression levels of the genes *HQT* (*hydroxycinnamoyl-CoA quinate hydroxycinnamoyl transferase*) Solycg07g005760 that is the central gene for the production of chlorogenic acid in tomato fruit (Moglia et al., 2014). Indeed, this gene catalyzes the formation of chlorogenic acid from caffeoyl CoA and quinic acid (Niggeweg et al., 2004). We also analyzed the expression levels of the gene Solyc01g079620 coding for the Myb12, a TF that regulates the production of flavonones and in particular of naringenin chalcone in tomato fruit (Ballester et al., 2010). We demonstrated that the gene *HQT* was down-regulated in IL7-3 and in IL12-4. A slightly higher expression for this gene was detected in DHO88-SL compared to M82. A drop in the expression of the gene coding for Myb12 was instead detected in the red ripe fruit of all the genotypes here tested compared to M82.

### Phylogenetic Analyses of Candidate Genes

**FIGURE 7:**
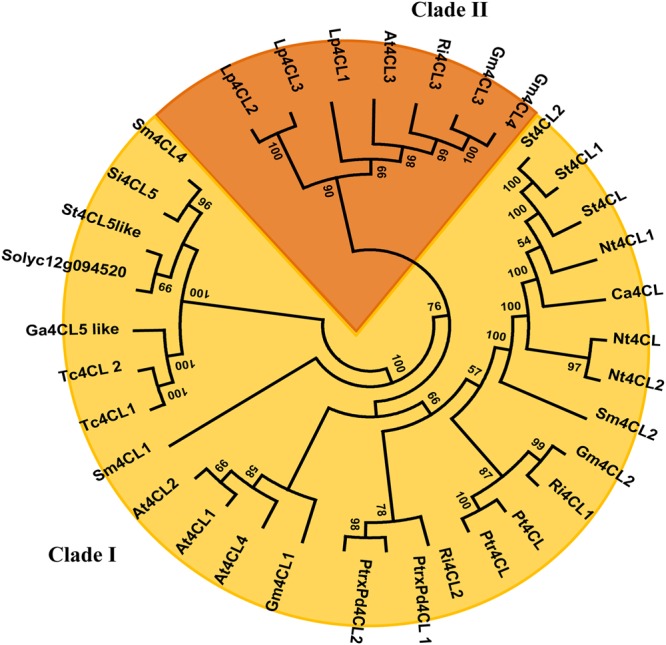
**Phylogenetic Tree of 4CL proteins.** The evolutionary history was inferred by using the Maximum Likelihood method based on the JTT matrix-based model. The tree with the highest log likelihood (-10913.5091) is shown. Initial trees for the heuristic search were obtained by applying the Neighbor-Joining method to a matrix of pairwise distances estimated using a JTT model. The analysis involved 34 amino acid sequences. All positions containing gaps and missing data were eliminated. There were a total of 444 positions in the final dataset. Evolutionary analyses were conducted in MEGA6. Branches corresponding to partitions reproduced in less than 50% bootstrap replicates are collapsed. The 4CL accession numbers are available in Supplementary Table S4.

Phylogenetic analyses were performed on the CGs *4CL* and *UGTs* identified in the introgressed regions 12-4 and 7-3. Since 4CL converts 4-coumaric acid and other cinammic acids (such as caffeic and ferulic acids) into corresponding CoA thiolesters then used for the biosynthesis of flavonoids, lignins, isoflavonoids, suberins, coumarins and wall-bound phenolics (Sun et al., 2013), members of the 4CL family have overlapping yet distinct roles in phenylpropanoid metabolism. A phylogenetic analysis of the 4CL superfamily was carried out exploiting the amino acid sequences of 34 4CL available from different plant species and allowed to generate a Maximum Likelihood (ML) tree. As shown in **Figure 7** class I and class II clades (Alberstein et al., 2012; Li et al., 2015) are distinctly defined, and Solyc12g094520 is closely linked to other 4CLs of class I, which have been previously associated with the biosynthesis of lignin and structurally related phenylpropanoid derivatives (Docimo et al., 2013). Instead, flavonoid biosynthesis has been mostly associated to Class II enzymes (Alberstein et al., 2012; Li et al., 2015). Therefore, albeit experimental studies are necessary for functional assignments, this preliminary analysis suggested that the tomato 4CL encoded by Solyc12g094520 could be mostly involved into channeling hydroxicinnamic acids into lignin synthesis rather than in flavonoid formation.

**FIGURE 8:**
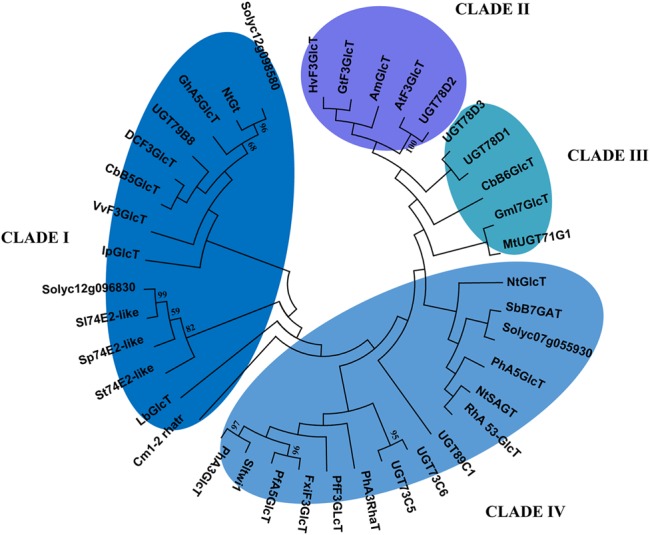
**Phylogenetic Tree of Flavonoid UGTs.** The evolutionary history was inferred by using the Maximum Likelihood method based on the JTT matrix-based model. The tree with the highest log likelihood (-42146.3982) is shown. Initial tree(s) for the heuristic search were obtained by applying the Neighbor-Joining method to a matrix of pairwise distances estimated using a JTT model. The analysis involved 38 amino acid sequences. All positions containing gaps and missing data were eliminated. There were a total of 402 positions in the final dataset. Evolutionary analyses were conducted in MEGA6. Branches corresponding to partitions reproduced in less than 50% bootstrap replicates are collapsed. The UGT accession numbers are available in Supplementary Table S4.

It is also known that the transfer from nucleoside diphosphateactivated sugars to aglycon substrates is catalyzed by glycosyl transferase enzymes; however, the substrate specificity of these enzymes includes several class of molecules, such as flavonoids, coumarins, terpenoids, and cyanohydrins (Shao et al., 2005). Therefore, since several UDP-glycosyltransferases mapped into the introgressed regions, we wanted to evaluate the relatedness of the identified tomato glycosyltransferases to other UGTs with different function. In order to predict a substrate specificity for the identified tomato UGT enzymes, a phylogenetic tree was constructed for Solyc07g055930, Solyc12g098580, and Solyc12g09683 along with other characterized UGTs from other plant families (**Figure 8**). The phylogenetic tree constructed on 39 UGT members highlighted the formation of four clusters. The two tomato glycosyltransferases from chromosome 12 clustered in the clade I, where mostly are grouped UGTs involved in the 3-*O*- and *5-O*-glycosilation of flavonoids, whereas clusters II and III mostly include enzymes characterized by flavonoid 5-*O*-glycosyltransferase and flavonoid 7-*O*-glycosyltransferase activity, respectively (Shao et al., 2005). Cluster III also contains glycosyltransferases that are unrelated to flavonoid biosynthesis and Cluster IV contains GTs that catalyze glycosyl transfer to sugar moieties of flavonoid glycosides. In this latter Cluster, the Solyc07g055930 was located more closely related to SbB7GAT an UGT involved in 7-*O*-glucuronosylation of baicalein in *Scutellaria baicalensis* and to PhA5GlcT, which is responsible for 5-*O*-glycosilation of anthocyanin in *Petunia hybrida.*

### Enzyme Activity in Pyramided Lines

**FIGURE 9:**
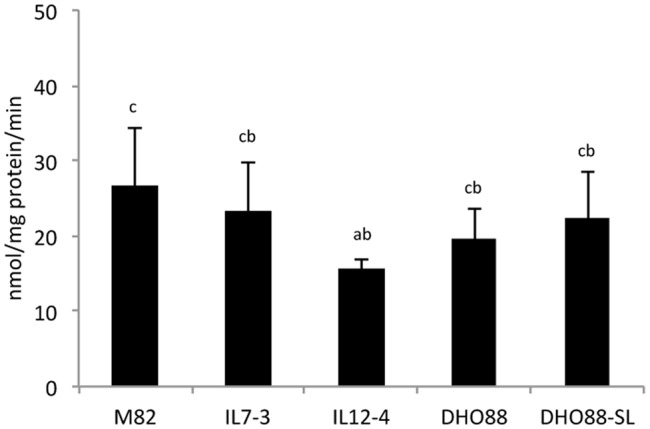
**Phenylalanine ammonia lyase (PAL) enzymatic activity in tomato lines: M82, IL7-3, IL12-4 and pyramided lines DHO88 and DHO88-SL.** Values expressed as nmol/mg protein/min are reported as mean ± SD of three biological replicates. Means denoted by the same letter do not differ significantly at *p* ≤ 0.05 according to Tukey HSD test.

Finally, we investigated the early steps of phenylpropanoid biosynthesis by measuring the enzymatic activities of PAL and 4CL enzymes in red ripe fruits of the tomato lines here analyzed. These analyses were performed in order to understand whether M82, the ILs and the pyramided lines showed a different ability to produce CoA activated molecules. We demonstrated that PAL activity was similar in M82, in IL7-3 and in the pyramided lines (**Figure 9**), whereas in the line IL12-4 the amount of cinnamoylCoA recorded was significantly lower than in M82.

**FIGURE 10:**
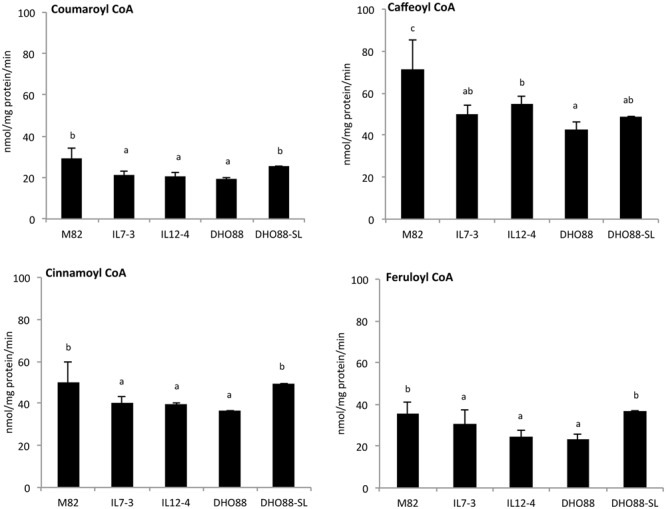
**4CL enzyme activity in M82, IL7-3, IL12-4, DHO88, and DHO88-SL. 4CL enzyme activities were measured toward *p*-coumaric acid, caffeic acid, cinnamic acid, and ferulic acid.** Values, expressed as nmol/mg protein/min, are reported as mean ± SD of three biological replicates. Means denoted by the same letter do not differ significantly at *p* ≤ 0.05 according to Tukey HSD test.

In addition, we assayed 4CL enzyme activity using four substrates: coumaric acid, ferulic acid, cinnamic acid, and caffeic acid (**Figure 10**). Overall, highest 4CL enzyme activity was found toward caffeic and ferulic acids, whereas a lower activity was detected for cinnamic and coumaric acids. Interestingly, for all the substrates, the 4CL enzyme activity recorded in IL7-3, IL12-4 and DHO88 was lower than the activity recorded in M82. On the contrary, the 4CL activity toward coumaric acid, ferulic acid, cinnamic acid was similar in M82 and in DHO88-SL. Only the 4CL activity toward caffeic acid was lower in DHO88-SL compared to M82.

## Discussion

Wild species are important sources of novel alleles for improving quality traits, such as antioxidant content, that could be introgressed into modern varieties by using traditional and innovative breeding approaches (Gur and Zamir, 2004, 2015; Schauer et al., 2006). In this regard, the production of ILs from wild species can help to facilitate the mapping of valuable traits originating from wild donors and to introduce unused alleles that were neglected during domestication (Gur and Zamir, 2015). Here, detailed analyses of metabolites accumulated in the fruit of two introgression lines (IL7-3 and IL12-4), of two pyramided lines obtained by crossing the two ILs (DHO88 and DHO88-SL) and of the cultivated line M82 were carried out. Metabolic analyses evidenced a lower content of flavonoids (naringenin glucoside and chalconaringenin) and phenolic acids (such as chlorogenic acid) in the red ripe fruits of both the introgression lines IL12-4 and IL7-3 compared to the control M82.

In the introgression lines IL7-3 the lower levels of phenylpropanoids detected were apparently caused by the down-regulation of one flavonoid biosynthetic gene, the *UGT* Solyc07g055930, and to the altered expression level of positive and negative regulators detected in the introgressed region 7-3. In particular, the lower expression of the Myb Solyc07g056120, putatively involved in the activation of the phenylpropanoid biosynthetic pathways, together with the higher expression of the Myb4-like Solyc07g053230, might have caused in the introgression line IL7-3 a block of the metabolic flux at the branch point represented by the 4-coumarate:CoA ligase. Several *Myb4-like* genes have been previously described as negative regulators of hydroxycinnamic acid biosynthesis in a group of plant species, directly repressing genes such as *cinnamate-4-hydrolase* and *4-coumarate:CoA ligase* (Perez-Diaz et al., 2016). Accordingly, in IL7-3 we detected a lower 4CL enzyme activity and a PAL activity that was comparable to that detected in M82. Moreover, in IL7-3 real time PCR demonstrated a lower expression of the gene *HQT*, a key gene for the biosynthesis of chlorogenic acid (Moglia et al., 2014), and of the TF Myb12, a TF that regulates the production of naringenin chalcone in the fruit (Ballester et al., 2010). These results further suggest a reduction in this IL of the flux through the hydroxycinnamate and flavonoid biosynthetic pathways. Moreover, the different expression levels observed for the *HQT* gene suggest the presence of regulatory proteins present in the introgression region that may control, directly or indirectly, the transcription of the *HQT* gene.

A lower level of phenolic acids and flavonoids was also detected in IL12-4 compared to the cultivated line M82. These results correlated well with the lower PAL and 4CL enzyme activity measured in this line compared to the cultivated line. These analyses indicate that in the introgression line IL12-4 a lower amount of precursors was available for chlorogenic acid, phenolic acids conjugated and flavonoids formation compared to M82. Accordingly, a lower expression level of the *HQT* gene and of the TF Myb12 was detected in IL12-4. Our hypothesis is that in the IL12-4 this altered metabolic flux was caused primarily by the down-regulation of one gene of the general phenylpropanoid pathway, the *4CL* gene identified in the upper part of the region 12-4. Additionally, the reduced flavonoid biosynthesis in IL12-4 may be caused by the down-regulation of the wild gene Solyc12g098580 coding for one UDP-glycosyltransferase and located in the lower part of the introgressed region 12-4. Phylogenetic analysis indicated that the protein encoded by this gene is closely related to GhA5GlcT and St74E2-like, and therefore possibly involved in 5-and 3-*O* glycosylation of flavonoids. In the fruits of several plant species (peach, apple, and grape), UDP-glucosyltransferase gene transcription is controlled by the involvement of different TFs, such as regulatory complexes composed by Myb, bHLH, and WD40 (Ravaglia et al., 2013). Two TFs, bHLH and WD40 (encoded by Solyc12g098620 and Solyc12g098690, respectively), putatively involved in regulating genes of the flavonoid pathway and located next to the gene Solyc12g098580 coding for the UDP-glucosyltransferase, were identified in the introgressed region 12-4. Interestingly, our transcriptional analyses demonstrated that these TFs were both down-regulated in IL12-4 and in both DHOs.

Considering the genetic background of IL7-3 and IL12-4, the pyramided lines DHO88 and DHO88-SL showed a peculiar accumulation of metabolites in their fruits. Indeed, in both the DHOs the content of phenolic acids increased, particularly the fraction of hexoses. In addition, a contrasting behavior was observed between the two different DHO genotypes here analyzed when the amount of free phenolic acids (such as chlorogenic acid) was considered. In particular, the line DHO88 exhibited a lower content of this fraction compared to M82, whereas the line DHO88-SL showed an accumulation level comparable to M82. These results are justified by the different size of the wild region carried on chromosome 12 in the two DHOs.

In the line DHO88, carrying the entire introgressions 7-3 and 12-4, we speculated that the lower levels of chlorogenic acid and flavonoids detected were primarily caused by the down-regulation of the wild *4CL* gene identified in IL12-4 and of the *UGTs* detected in both ILs. The lower amount of phenylpropanoids detected in this line was likely also due to the influence of regulatory protein coded by genes present in both the introgressed regions. Surprisingly, in DHO88, which carries the wild *4CL*, we could not detect any differences in the expression levels of the *HQT* gene compared to M82 and the PAL enzyme activity was not altered. However, a lower expression level of the TF Myb12 was demonstrated and a lower 4CL enzyme activity was also recorded in this line toward all the substrates tested. The phylogenetic study carried out indicated that the 4CL isoform encoded by the Solyc12g094520 could be mostly involved into channeling hydroxycinnamic derivatives for lignin formation (Alberstein et al., 2012; Sun et al., 2013). Indeed, the 4CL isoform here identified clustered with type I 4CLs, as well as the 4CL identified in *Salvia miltiorrhiza* (Sm4CL4, accession number AGW27194), which are reported to be involved in lignin biosynthesis (Alberstein et al., 2012; Sun et al., 2013).

Therefore, the higher levels of phenolic acid hexose detected in DHO88 could indicate that the pool of precursors left unused by the flavonoid biosynthetic pathway and also by the lignin biosynthetic pathway had been reallocated to the synthesis of other phenolic compounds. Indeed, recent work carried out in *Arabidopsis thaliana* and in tomato demonstrated that, if downstream branches of the phenylpropanoid pathway are less active, this could lead to the reorientation of the carbon flux with a consequent accumulation of various classes of hexosylated phenylpropanoids (van der Rest et al., 2006; Vanholme et al., 2012).

In the line DHO88-SL, carrying the entire introgression 7-3 and the lower part of the introgression region 12-4, a reduced content of flavonoids (rutin, naringenin glucoside, and chalconaringenin) was also found compared to M82. The lower expression level of the *UGTs* Solyc12g098580 and Solyc07g055930, together with the additional influence of regulatory proteins present in both the introgressed regions 7-3 and 12-4, might reduce the levels of flavonoids detected. As expected, the expression of the TFs Myb12 was lower in DHO88-SL compared to M82. Interestingly, the level of phenolic acids hexoses was higher in DHO88-SL compared to the parental lines IL7-3 and IL12-4 and also to the pyramided line DHO88. In addition a higher level of chlorogenic acid compared to the parental lines and to the pyramided line DHO88 was demonstrated. These results correlated well with the results obtained with the biochemical analyses that demonstrated that the 4CL activity toward coumaric acid, ferulic acid and cinnamic acid was similar in DHO88-SL compared to M82 and was higher compared to the parental lines and to DHO88. Accordingly, real-time PCR analyses demonstrated that the expression level of the gene *HQT* was slightly higher in DHO88-SL compared to M82. Therefore we concluded that in the pyramided line DHO88-SL, that carries the cultivated allele for *4CL* in the homozygous state, a major accumulation of cinnamic acid intermediates remained available for hexose conjugation but also for chlorogenic acid formation, thus indicating the presence of an enzymatic machinery correctly working (van der Rest et al., 2006; Vanholme et al., 2012). This result confirmed the central role of the *4CL* gene identified in IL12-4 in the redirection of the phenylpropanoid biosynthetic pathways in the pyramided lines DHO88 and DHO88-SL.

Hydroxycinnamates that accumulated in high amount in the lines here described have several beneficial health activity including very potent antioxidant activity and hepatoprotective, hypoglycaemic and antiviral activities (Tohge et al., 2015). Consequently, there is an increasing interest in the production of alternative dietary sources that are rich in these phenolic compounds (Tohge et al., 2015). Results obtained in this study suggest that pathway rerouting may be a valid strategy in order to produce tomatoes with a higher amount of hydroxycinnamic acids in the fruit. Altogether, results obtained in this work highlighted that, in order to design an efficient pyramiding strategy for increasing tomato nutritional quality, detailed information on the possible interaction effects between QTLs are necessary.

## Conclusion

Here, we integrated genomic, transcriptomic and biochemical analyses to identify CGs controlling phenylpropanoid accumulation in the fruits of pyramided lines obtained by crossing two *S. pennellii* introgression lines (IL12-4 and IL7-3). One pyramided genotype (DHO88-SL) was demonstrated to contain a higher amount of phenolic acids and phenolic acids hexose in the fruits compared to the parental lines. This increase was due to changes in the formation and/or availability of compounds in the different branches of the phenylpropanoid biosynthetic pathway caused by the combined effects of the two introgressed regions 12-4 and 7-3. In fact, a repression of flavonoid synthesis in the pyramided line DHO88-SL was accompanied by an increased synthesis of products from other branches of the phenylpropanoid pathway such as caffeic acid hexose. Moreover, analyses carried out in this paper highlighted the central role of one 4-coumarate:CoA ligase identified in the region 12-4, in the perturbation of the phenylpropanoid biosynthetic pathways in the pyramided lines DHO88 and DHO88-SL. Now, experiments involving reverse genetic approaches are underway in order to unveil the functional role of the CGs here detected to better define their role in tomato fruits.

## Author Contributions

MR, AR, TD, VR contributed to metabolic, biochemical and transcriptomic analyses, to the experimental analyses carried out to identify CGs, and to draft the manuscript; RC contributed to molecular marker analysis and to grow materials; PV and RF contributed to metabolic analysis and critically revised the manuscript; LF contributed to the conception of the experiment and critically revised the manuscript; AB contributed to the experiment design, to data analysis and interpretation, to draft the manuscript.

## Conflict of Interest Statement

The authors declare that the research was conducted in the absence of any commercial or financial relationships that could be construed as a potential conflict of interest.

## Footnotes

1 http://tgrc.ucdavis.edu/

2 www.ted.bti.cornell.edu
