# Supplementary material for: Metabolic and Molecular Changes of the Phenylpropanoid Pathway in Tomato (Solanum lycopersicum) Lines Carrying Different Solanum pennellii Wild Chromosomal Regions
Source: Front Plant Sci. 2016 Oct 4;7:1484. doi: 10.3389/fpls.2016.01484 (PMC5047917; doi:10.3389/fpls.2016.01484)
Supplement: Supplementary file 1 [file Data_Sheet_1.docx]

Supplementary Material

**Metabolic and Molecular Changes of the Phenylpropanoid Pathway in Tomato (*Solanum lycopersicum*) Lines Carrying Different *Solanum pennellii* Wild Chromosomal Regions**

**Maria Manuela Rigano^1^, Assunta Raiola^1^, Teresa Docimo^2^, Valentino Ruggieri^1^, Roberta Calafiore^1^, Paola Vitaglione^1^, Rosalia Ferracane^1^, Luigi Frusciante^1^, Amalia Barone^1^**^*^

^1^Department of Agricultural Sciences, University of Naples Federico II, Portici (Naples), Italy

^2^Istituto di Bioscienze e BioRisorse, UOS Portici, Consiglio Nazionale delle Ricerche, Portici (Naples), Italy

*** Correspondence:**Amalia Barone,

ambarone@unina.it

# Supplementary Figures and Tables

**1.1. Supplementary Tables**

Supplementary Table S1: Physico-chemical properties of the soil at Acerra (Naples, Italy).

| Sand | 58.8 mg/100 g |
| --- | --- |
| Loam | 30.9 mg/100 g |
| Clay | 10.3 mg/100 g |
| Soil Organic Matter | 20.2 mg/1000 g |
| pH | 7.3 |
| Total N | 1.4 g/1000 g |
| C:N ratio | 8.2 |
| Cation exchange capacity | 20 meq/100 g |
| Exchangeable calcium | 13.79 meq/100 g |
| Exchangeable magnesium | 0.94 meq/100 g |
| Exchangeable sodium | 0.69 meq/100 g |
| Exchangeable potassium | 4.55 meq/100 g |
| Available phosphate | 216 ppm (P_2_O_5_) |
| Available potassium | 2173 ppm (K_2_O) |
| Conductivity 1:5 25°C | 0.07 dS/m |

**Supplementary Table S2:** MS/MS characteristics of phenolic compounds identified in tomato fruit extracts.

| **Compound** | **[M-H]^-^ *m/z*** | **Product ions**  **[M-H]^-^ *m/z*** |
| --- | --- | --- |
| Rutin pentoside | 741 | 609 |
| Kaempferol rutinoside | 593 | 285 |
| Rutin | 609 | 301; 271 |
| Naringenin hexoside | 433 | 271 |
| Naringenin glucoside | 433 | 271 |
| Naringenin diglycoside | 595 | 385; 355 |
| Naringenin chalcone | 271 | 119; 151 |
| Quercetin | 301 | 151; 179 |
| Coumaric acid-glucoside | 325 | 163 |
| Chlorogenic acid | 353 | 191 |
| Caffeic acid | 179 | 135 |
| Ferulic acid glucoside | 355 | 193 |
| *p-*Coumaric acid | 163 | 119; 113 |
| Caffeic acid glucoside | 341 | 179 |
| Gallic acid | 169 | 125; 79 |
| Protocatechuic acid | 153 | 109 |
| Protocatechuic acid hexoside | 315 | 153 |

**Supplementary Table S3:** Sequences of adopted primers for amplifying by Real-Time qPCR selected candidate genes and the housekeeping gene *EF-1α*.

| **Primer** | **Sequence** |
| --- | --- |
| Solyc01g079620FW | 5’-AGC TTG TGA TAG TGC CAT GG-3’ |
| Solyc01g079620REV | 5’-CAC TAT CTG AAC TAA GGC TTC CC-3’ |
| Solyc06g005060FW | 5’-CAACCCTGACAAAATCCCCTTT-3’ |
| Solyc06g005060RV | 5’-TTGGTCCCTTGTACCAGTCGAG-3’ |
| Solyc07g005760FW | 5’-CCA CCT ACC CTA AAC TCA TCG-3’ |
| Solyc07g005760REV | 5’-GGG CTG CGA GGA TTT CAT AG-3’ |
| Solyc07g062030FW | 5’-TTG GTG TCA AGG TCT ATG CTG-3’ |
| Solyc07g062030REV | 5’-CTC CAA ATC CGC TTC AAA GAT C-3’ |
| Solyc07g053230FW | 5’-GGA ATT GGA GAC AAC TAC CCA-3’ |
| Solyc07g053230REV | 5’-TTG AGC ATG GAG GTT CAA GA-3’ |
| Solyc07g055930FW | 5’-GTT GGA TTA TGA TCG TTG CG-3’ |
| Solyc07g055930REV | 5’-TTC CAC AAT TCG AAA CCA GA-3’ |
| Solyc07g056120FW | 5’-GTG ATC AAC AAT GGG TGG TT-3’ |
| Solyc07g056120REV | 5’-TTA CCT GAA TGC CAT TTC CA-3’ |
| Solyc12g094520 FW | 5’-CGA GCA TGG AAG GGA AAA TTG-3’ |
| Solyc12g094520 REV | 5’-TCA GAG TCT AGA GTG GAA GCA G-3’ |
| Solyc12g096830FW | 5’-CAT TTG ATG TGT TGG AGA AGG AG-3’ |
| Solyc12g096830REV | 5’-ATG GAA TAA GTG GAC CGA TGG-3’ |
| Solyc12g098580FW | 5’-GGT TGG ATT CGA GAC ATG AGG-3’ |
| Solyc12g098580REV | 5’-CTT CGT CTT CCC CTT TTC TCC-3’ |
| Solyc12g098620 FW | 5’- GAT TAT ATT CAT GTT AGA GCT AGA AGG G – 3’ |
| Solyc12g098620 REV | 5’- CAT CAC AGC TTT TCC AGT CAC -3’ |
| Solyc12g098690FW | 5’- CGA ATT TGG GAC ATG AGA AAC C -3’ |
| Solyc12g098690REV | 5’- GAG TAA CGG ATT GAT CGG ACA G-3’ |

**Supplementary Table S4:** List of 4-Coumarate:CoA ligase and Glucosyltransferase proteins used to build the phylogenetic trees described in **Figure 7** and **Figure 8**.

| Species | Protein symbol | Protein | Accession  number |
| --- | --- | --- | --- |
| 4-Coumarate:CoA ligases | | | |
| *Arabidopsis thaliana* | At4CL1 | 4-coumarate:CoA ligase 1 | AAM20598 |
| *Arabidopsis thaliana* | At4CL2 | 4-coumarate:CoA ligase 2 | AAD47193 |
| *Arabidopsis thaliana* | At4CL3 | 4-coumarate:CoA ligase 3 | AAD47195 |
| *Arabidopsis thaliana* | At4CL4 | 4-coumarate:CoA ligase 4 | Q9LU36 |
| *Capsicum annuum* | Ca4CL | 4-coumarate:CoA ligase | AAG43823 |
| *Glycine max* | Gm4CL1 | 4-coumarate:CoA ligase 1 | AAL98709 |
| *Glycine max* | Gm4CL2 | 4-coumarate:CoA ligase 2 | AAC97600 |
| *Glycine max* | Gm4CL3 | 4-coumarate:CoA ligase 3 | AAC97599 |
| *Gossypium arboreum* | Ga4CL-like5 | 4-coumarate:CoA ligase-like 5 | KHG05050 |
| *Lolium perenne* | Lp4CL1 | 4-coumarate:CoA ligase 1 | AAF37732 |
| *Lolium perenne* | Lp4CL2 | 4-coumarate:CoA ligase 2 | AAF37733 |
| *Lolium perenne* | Lp4CL3 | 4-coumarate:CoA ligase 3 | AAF37734 |
| *Nicotiana tabacum* | Nt4CL | 4-coumarate:CoA ligase | AAB18638 |
| *P trichocarpa x P. deltoides* | Pt xPd4CL1 | 4-coumarate:CoA ligase 1 | AAC39366 |
| *P trichocarpa x P. deltoides* | Pt xPd4CL2 | 4-coumarate:CoA ligase 2 | AAC39365 |
| *Populus tomentosa* | Pt4Cl | 4-coumarate:CoA ligase | AAL56850 |
| *Populus tomentosa* | Pt4CL | 4-coumarate:CoA ligase | AAL02145 |
| *Populus tremuloides* | Ptr4CL | 4-coumarate:CoA ligase | AAC24503 |
| *Rubus idaeus* | Ri4CL1 | 4-coumarate:CoA ligase 1 | AAF91310 |
| *Rubus idaeus* | Ri4CL2 | 4-coumarate:CoA ligase 2 | AAF91309 |
| *Rubus idaeus* | Ri4CL3 | 4-coumarate:CoA ligase 3 | AAF91308 |
| *Salvia miltiorrhiza* | Sm4CL1 | 4-coumarate:CoA ligase 1 | AAP68990 |
| *Salvia miltiorrhiza* | Sm4CL2 | 4-coumarate:CoA ligase 2 | AAP68991 |
| *Salvia miltiorrhiza* | Sm4CL4 | 4-coumarate:CoA ligase 4 | AGW27194 |
| *Sesamum indicum* | Si4CL-like 5 | 4-coumarate:CoA ligase-like 5 | XP_011099557 |
| *Solanum tuberosum* | St4CL1 | 4-coumarate:CoA ligase 1 | P31684 |
| *Solanum tuberosum* | St4CL2 | 4-coumarate:CoA ligase 2 | P31685 |
| *Solanum tuberosum* | St4CL | 4-coumarate:Co A ligase | AAD40664 |
| *Solanum tuberosum* | St4CLlike5 | 4-coumarate:CoA ligase-like 5 | XP_006342547 |
| *Theobroma cacao* | Tc4CL1 | 4-coumarate:CoA ligase 1 | XP_007017972 |
| *Theobroma cacao* | Tc4CL2 | 4-coumarate:CoA ligase 2 | XP_007017973 |
| Glucosyltransferases | | | |
| *Antirrhinum majus* | AmC4GT | Chalcone 4'-O-glucosyltransferase | Q33DV3 |
| *Antirrhinum majus* | UGT73E2 | Flavonoid glucoyltransferase | BAG16513 |
| *Arabidopsis thaliana* | AtF3GT | Flavonoid 3-O-glucosyltransferase | AAM91139 |
| *Arabidopsis thaliana* | At73B1 | UDP-glucosyltransferase 73B1 | NP_567955 |
| *Arabidopsis thaliana* | UGT89C1 | UDP-glycosyltransferase 89C1 | NP_563756 |
| *Arabidopsis thaliana* | UGT79B1 | Anthocyanidin 3-O-glucoside  2'''-O-xylosyltransferase | Q9LVW3 |
| *Arabidopsis thaliana* | UGT79B8 | UDP-glycosyltransferase 79B8 | O81010 |
| *Arabidopsis thaliana* | UGT73C6 | UDP-glycosyltransferase 73C6 | NP_181217 |
| *Arabidopsis thaliana* | UGT89C1 | Flavonol 7-O-rhamnosyltransferase | Q9LNE6 |
| *Citrus maxima* | Cm12RhaT | UDP-rhamnose:flavanone-7-O-glucoside-2-O-rhamnosyltransferase | Q8GVE3 |
| *Cleretum bellidiforme* | CbB5GlcT | Betanidin-5-O-glucosyltransferase | CAB56231 |
| *Cleretum bellidiforme* | CbB6GlcT | Betanidin 6-O-glucosyltransferase | AAL57240 |
| *Dianthus caryophyllus* | DcF3GlcT | UDP-glucose : flavonol 3-O-glucosyltransferase | BAD52004 |
| *Forsythia x intermedia* | FxiF3GLcT | Flavonoid 3-O-glucosyltransferase | AAD21086 |
| *Gentiana triflora* | GtA3GT | Anthocyanidin 3-O-glucosyltransferase | Q96493 |
| *Glandularia x hybrida* | GhA5GlcT | UDP-glucose : anthocyanin 5-O-glucosyltransferase | BAA36423 |
| *Glycine max* | GmI7GlcT | Isoflavone 7-O-glucosyltransferase 1 | NP_001235161 |
| *Glycyrrhiza echinata* | GeIGlcT | Isoflavonoid glucosyltransferase | BAC78438 |
| *Hordeum vulgaris* | HvF3GT | Flavonoid 3-O-glucosyltransferase | P14726 |
| *Ipomoea purpurea* | IpGlcT | Glucosyltransferase | BAD95882 |
| *Lycium barbarum* | LbGlcT | Putative glycosyltransferase | BAG80534 |
| *Nicotiana tabacum* | NtGT | Glucosyltransferase | BAB88935 |
| *Nicotiana tabacum* | NtSAGT | UDP-glucose:salicylic acid glucosyltransferase | AAF61647 |
| *Nicotiana tabacum* | NtGlcT | Glucosyltransferase | BAB88934 |
| *Perilla frutescens* | PfA5GlcT | UDP-glucose : anthocyanin 5-O-glucosyltransferase | Q9ZR27 |
| *Perilla frutescens* | PfF3GlcT | Flavonoid 3-O-glucosyltransferase | BAA19659 |
| *Petunia x hybrida* | PhA5GlcT | Anthocyanin 5-O-glucosyltransferase | BAA89009 |
| *Petunia x hybrida* | PhA | Anthocyanin 3 glucoside: rhamnosyltransferase | CAA50376 |
| *Rosa hybrid cultivar* | RhA5,3GlcT | UDP-glucose: anthocyanidin 5,3-O-glucosyltransferase | BAD99560 |
| *Scutellaria baicalensis* | SbB7GAT | UDP-glucuronate:baicalein 7-O-glucuronosyltransferase | Q76MR7 |
| *Solanum lycopersicum* | Sl74E2like | UDP-glycosyltransferase 74E2-like | XP_004253027 |
| *Solanum lycopersicum* | SlTwi | Twi1partial | CAA59450. |
| *Solanum pennellii* | Sp74E2like | UDP-glycosyltransferase 74E2-like | XP_015059609 |
| *Solanum tuberosum* | St74E2like | UDP-glycosyltransferase 74E2-like | XP_006342417 |
| *Verbena hybrida* | VhA5GT | UDP-glucose:anthocyanin 5-O-glucosyltransferase | Q9ZR25 |
| *Vitis vinifera* | VvF3GlcT | Flavonoid 3-O-glucosyltransferase | 88192533 |

**Supplementary Table S5**: Quantification of single identified phenolic compounds by LC/MS/MS

| **Phenolic compounds** | **M82** | **IL7-3** | **IL12-4** | **DHO88** | **DHO88-SL** |
| --- | --- | --- | --- | --- | --- |
| Chlorogenic acid | 8.16±0.34^b^ | 4.60±0.27^a^ | 4.85±0.14^a^ | 4.77±0.19^a^ | 7.73±0.45^b^ |
| Ferulic acid hexose | 0.10±0.01^b^ | 0.05±0.01^a^ | 0.05±0.01^a^ | 0.05±0.01^a^ | 0.08±0.01^b^ |
| Coumaric acid hexose | 2.05±0.12^b^ | 1.87±0.25^ab^ | 1.62±0.22^a^ | 2.48±0.01^c^ | 2.56±0.31^c^ |
| Protocatechuic acid hexose | <LOQ | <LOQ | <LOQ | <LOQ | <LOQ |
| Caffeic acid hexose | 1.79±0.26^a^ | 1.74±0.09^a^ | 1.71±0.07^a^ | 2.18±0.13^b^ | 2.71±0.23^c^ |
| Caffeic acid | 0.16±0.01^b^ | 0.14±0.01^b^ | 0.11±0.01^a^ | 0.14±0.01^b^ | 0.24±0.01^c^ |
| Coumaric acid | <LOQ | <LOQ | <LOQ | <LOQ | <LOQ |
| Gallic acid | <LOQ | <LOQ | <LOQ | <LOQ | <LOQ |
| Kaempferol rutinoside | <LOQ | <LOQ | <LOQ | <LOQ | <LOQ |
| Pentosil rutin | 0.02±0.01^a^ | 0.02±0.01^a^ | 0.01±0.01^a^ | 0.02±0.01^a^ | 0.01±0.01^a^ |
| Rutin | 2.00±0.25^b^ | 1.66±0.09^b^ | 1.13±0.13^a^ | 1.84±0.06^b^ | 1.14±0.25^a^ |
| Naringenin glucoside | 2.80±0.21^d^ | 1.40±0.10^c^ | 0.80±0.17^a^ | 1.08±0.02^b^ | 0.71±0.07^a^ |
| Naringenin diglucoside | 0.06±0.01^b^ | 0.05±0.01^a^ | 0.05±0,01^a^ | 0.05±0.01^a^ | 0.05±0.01^a^ |
| Chalcone naringenin | 12.77±0.61^b^ | 6.53±0.01^a^ | 5.85±1.07^a^ | 7.47±1.42^a^ | 6.02±0.42^a^ |
| Quercetin | 0.02±0.01^a^ | 0.02±0.01^a^ | 0.01±0.01^a^ | 0.02±0.01^a^ | 0.01±0.01^a^ |

## Supplementary Figures


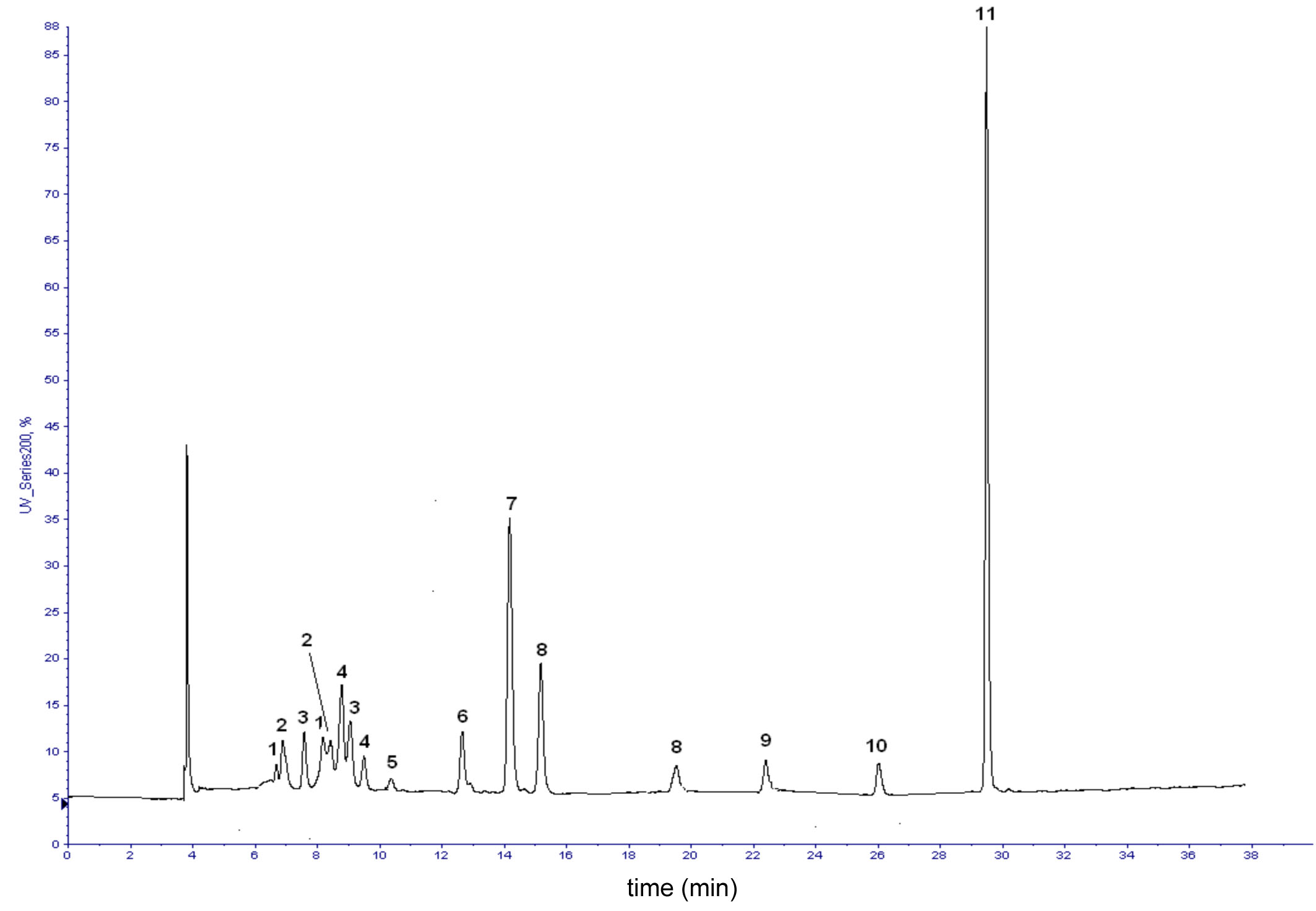


**Supplementary Figure S1.** Chromatogram of phenolic compounds detected at 330 nm in the line M82. Peaks:1: Caffeic acid hexose; 2: Coumaric acid hexose; 3: Ferulic acid glucoside; 4: Chlorogenic acid; 5: Caffeic acid; 6: Rutin pentoside; 7: Rutin; 8: Naringenin glucoside; 9: Naringenin hexoside; 10: Quercetin; 11: Chalcone naringenin. The peaks with the same number are isomers.
